# Clinical efficacy and safety of mesenchymal stem cell transplantation for osteoarthritis treatment: A meta-analysis

**DOI:** 10.1371/journal.pone.0175449

**Published:** 2017-04-27

**Authors:** Ma Yubo, Li Yanyan, Li Li, Sun Tao, Lin Bo, Chen Lin

**Affiliations:** 1 Department of Orthopaedics, Hongqi Hospital, Mudanjiang Medical University, Mudanjiang City, Heilongjiang Province, China; 2 Department of Neurology, The Second People Hospital of Mudanjiang, Mudanjiang City, Heilongjiang Province, China; 3 Department of Basic Medicine, Mudanjiang Medical University, Mudanjiang City, Heilongjiang Province, China; 4 Department of Radiology, Hongqi Hospital, Mudanjiang Medical University, Mudanjiang City, Heilongjiang Province, China; 5 Department of Orthopaedics, The 2nd Affiliated Hospital of Harbin Medical University, Harbin City, Heilongjiang Province, China; Cardiff University, UNITED KINGDOM

## Abstract

**Purpose:**

The aim of this study was to evaluate the therapeutic efficacy and safety of mesenchymal stem cells (MSCs) for the treatment of patients with knee osteoarthritis (OA).

**Materials:**

We performed a meta-analysis of relevant published clinical studies. An electronic search was conducted for randomized controlled trials (RCTs) of MSC-based therapy in knee OA. The visual analogue scale (VAS), International Knee Documentation Committee (IKDC) form, Western Ontario and McMaster Universities Osteoarthritis Index (WOMAC), Lequesne algofunctional indices (Lequesne), Lysholm knee scale (Lysholm), Tegner activity scale (Tegner) and adverse events (AEs) were evaluated.

**Results:**

Eleven eligible trials with 582 knee OA patients were included in the present meta-analysis. We demonstrated that MSC treatment could significantly decrease VAS and increase IKDC scoresafter a 24-month follow-up compared with controls (*P*<0.05). MSC therapy also showed significant decreases in WOMAC and Lequesne scores after the 12-month follow-up (*P*<0.01). Analysis of Lysholm (24-month) and Tegner (12- and 24-month) scores also demonstrated favorable results for MSC treatment (*P*<0.05).

**Conclusion:**

Overall, MSC transplantation treatment was shown to be safe and has great potential as an efficacious clinical therapy for patients with knee OA.

## 1. Introduction

The knee is a marvel of engineering that enables sophisticated movements and also acts as a conduit for transferring body weight in a way that is essential for normal human mobility [1,2]. Knee osteoarthritis(OA) is a chronic disease which affects all races, genders and ages but is known to be most in obese and in elderly people [3]. Knee OA includes self-reported knee OA, radiographic definitions of knee OA, and symptomatic knee OA (self-reported joint pain, stiffness, tenderness, and radiographic evidence) [4]. The menisci are known to maintain the normal function of the knee, distribute loads, lubricate the joint, and facilitate joint stability [5–7]. In general, partial or total meniscectomy causes OA of the knee [8,9]. Worldwide, arthritis is considered to be the fourth leading cause of disability [10,11]. In developing and developed countries, OA may cause a significant decline in the quality of life for individuals above the age of 65 due to joint pain and disability [2,12–15].

The basic pathophysiological characteristic of OA is a loss of articular cartilage, although the synovial membrane, bone or other components of the joint may also be affected [2,16–18]. Chondrocytes are the main component of the cartilage. These cells are relatively inert, and rarely regenerate [13–15]. The outer third of the meniscus (also known as the red-red zone) has better self-healing capabilities compared with other regions due to a good blood supply. Conventional therapies for OA include physiotherapy, anti-inflammatory drugs, pain-relieving drugs, hyaluronic acid, platelet-rich plasma or corticosteroid-based intra-articular injections, and knee arthroscopic surgery [19–21]. Unfortunately, these treatments have demonstrated modest clinical benefits compared with controls, and articular replacement by prosthesis is recommended as a last therapeutic option [2,3,5].

Medical researchers believe that tissue engineering, an innovative and effective therapy method, is the next logical step in the progression of surgical intervention [5,22,23]. There are three main types of cells used in the clinical trials for knee OA or degenerative conditions, including mesenchymal stem cells (MSCs), articular chondrocytes, and meniscal fibrochondrocytes (MFCs). Among the various cell therapies, MSC therapies are promising for the treatment of OA and have shown encouraging results. Clinicaltrials.gov lists 125 registered trials of knee OA with the key words of “MSCs” and “knee osteoarthritis”until October 2016, including umbilical cord-derived mesenchymal stem cells (UCMSCs), bone marrow-derived mesenchymal stem cells (BMSCs), adipose-derived stem cells (ADSCs), synovium-derived mesenchymal stem cells (SMSCs), and meniscus-derived mesenchymal stem cells (MeMSCs). In 2011, Cupistem (Anterogen) was approved by the Korean Food and Drug Administration (FDA) for the treatment of OA, and UCMSCs were the main ingredient of this drug.

In this study, we performed a systematic review and meta-analysis of randomized controlled trials (RCTs) to assess the efficacy and safety of MSC-based stem cell therapy in knee OA treatment and to provide additional treatment options for patients with knee OA. The goal of the present study was to evaluate the clinical response to MSC-based stem cell therapy by using the Lysholm knee scale (Lysholm), Tegner activity scale (Tegner), visual analogue scale (VAS), International Knee Documentation Committee (IKDC) form, Western Ontario and McMaster Universities Osteoarthritis Index (WOMAC), Lequesne algofunctional indices (Lequesne), and adverse events (AEs).

## 2. Materials and methods

### 2.1. Search strategy, study design, and eligibility criteria

Science Direct, Springer-Link, PubMed, the Wangfang Database, the China Science and Technology Journal Database, and China Journal Net were searched for the relevant studies published from 1980 to October, 2016. The search strategy included the keywords (“mesenchymal stem cells” OR “MSCs”) AND (“knee osteoarthritis” OR “knee articular cartilage regeneration” OR “knee cartilage defect”) AND clinical trial, without language restriction. We also searched the Clinicaltrials.gov for information on ongoing trials, using the keywords (“MSCs”) AND (“knee osteoarthritis”). Publication citations were displayed at the bottom of the “Full Text View” tab of a study record, under the “More Information” heading. Furthermore, previously published clinical trials, relevant review articles, and postgraduate papers were examined to identify further relevant studies. Studies were eligible for inclusion if: (1) they were published RCTs in humans of MSC transplantation therapy for patients with knee OA, (2) the patient’s detailed information was reported both prior to and after therapy, and (3) the study enrolled 10 or more patients. Phase IMSC-based stem cell therapy trials and review studies were excluded. In addition, case reports, studies on animal models and cell lines, and studies with no appropriate control arm were excluded.

### 2.2. Data selection criteria and quality assessment

Study selection and data extraction were independently conducted by two reviewers (Li Yanyan and Li Li) using a standardized approach. Any differences were adjudicated by a third reviewer (Ma Yubo) after referring back to the original publication. The extracted study data features included the first author name, year and country of publication, clinical trial phase, sample size per arm, mean patient age, previous treatments, follow-up time, and dose and route of MSCs administration. The overall quality of each included paper was evaluated by the Jadad scale [24]. Several major criteria were employed in a grading scheme: (1) randomization, (2) allocation concealment, (3) blinding, (4) lost to follow up, (5) intention to treat (ITT), and (6) baseline.

### 2.3. Definition of outcome measures

VAS improvement was defined as the mean change in VAS from baseline. IKDC and WOMAC improvement were defined as the mean changes in IKDC and WOMAC from baseline, respectively. Lequesne reduction was defined as the mean change in Lequesne from baseline. The primary outcome measures were absolute change in VAS, IKDC, WOMAC, and Lequesne. Lysholm and Tegner improvement were defined as the mean changes in Lysholm and Tegner from baseline, respectively. Secondary outcome measures were absolute change in Tegner and Lysholm clinical scores.

### 2.4 Statistical analysis

In this meta-analysis, we compared the MSC treatment groups from the identified trials with their respective control groups using Review Manager Version 5.0 (Nordic Cochran Centre, Copenhagen, Denmark). Heterogeneity among the trials was assessed with the *χ*^*2*^-based Q-test and the *I*^*2*^ statistic, such that *I*^*2*^>50% was considered to indicate a high level of heterogeneity. Fixed- and random-effects models were used to estimate MSC treatment effects. Afixed-effects model was used when statistical heterogeneity was not confirmed; otherwise, a random-effects model was employed. The MSC treatment effects were reflected by the mean differences (MDs) with 95% confidence intervals (CIs). *P*≤0.05 was considered to be statistically significant in all analyses, and all reported *P*-values resulted from two-sided version tests of the respective tests. To assess the possibility of publication bias, Egger’s test and Begg’s test were used (Stata version12.0, Stata Corporation, USA). We also used a funnel plot to evaluate publication bias.

## 3. Results

### 3.1. Trial selection

**Fig 1:**
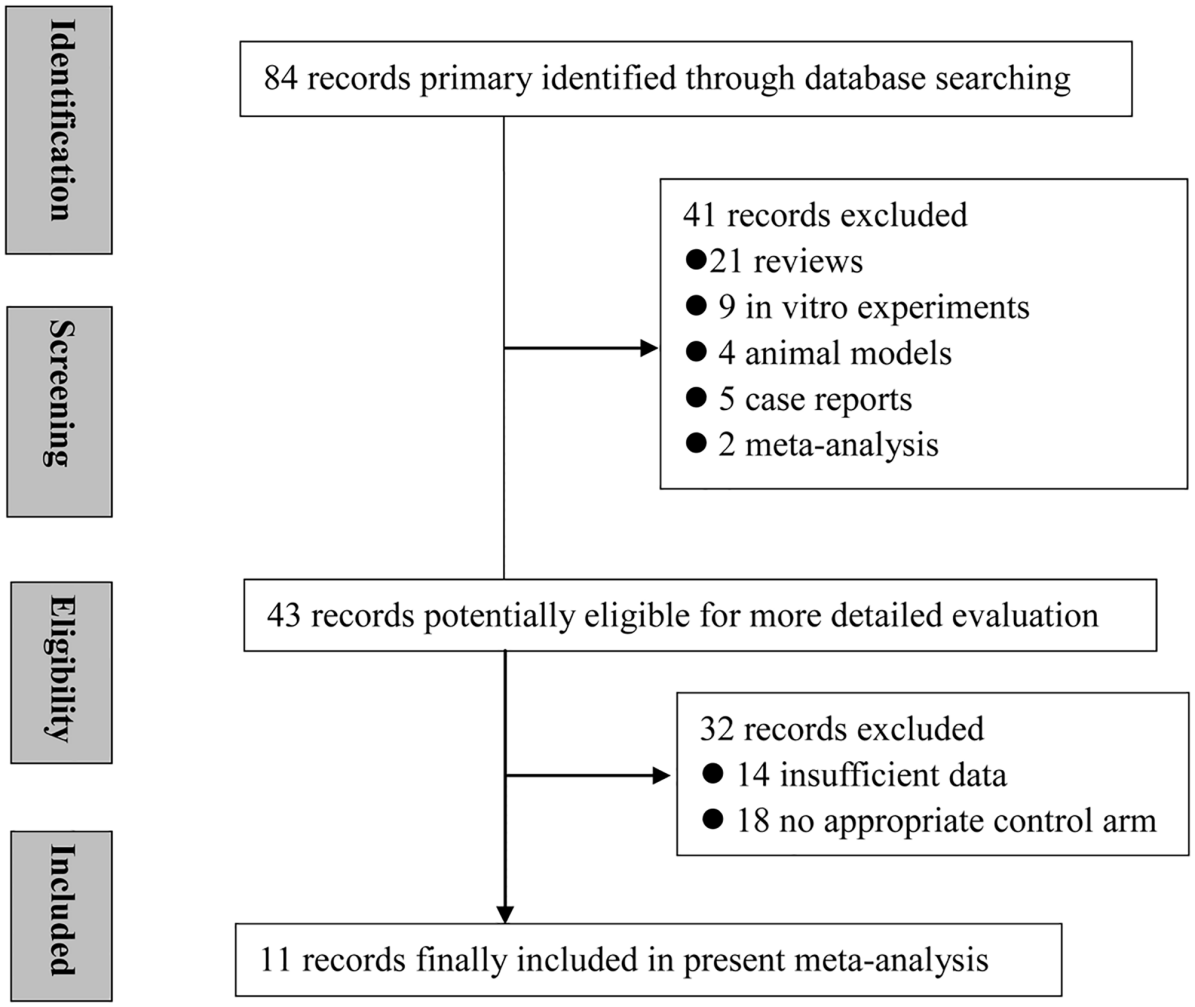
Flow diagram showing the study identification, screening, and inclusion process.

The data search yielded 84 references, 41 of which were excluded for various reasons (21 review articles, 13 in vitro experiments or animal models, 5 case reports, and 2 meta-analyses). A further 32 studies were excluded because they did not provide clinical data with enough detail or they were not RCTs. Finally, 11 trials met the specified inclusion criteria [25–35]. Fig 1 provides a flow-chart illustrating the search results and the exclusion mechanisms for certain studies. The quality assessment of the 11 trials is summarized in Table 1. Six of the included studies scored an A on the Jadad scale [26,27,28,30,31,32], and fivescored a B [25,29,33,34,35]. The funnel plots for the six analyses regarding VAS, IKDC, WOMAC, Lequesne, Lysholm, and Tegner were largely symmetrical (S1 Fig). Egger’s test and Begg’s test showed that there was no evidence of publication bias (*P*>0.05). Thus, publication bias did not seem to be present in our study.

**Table 1:** Jadad scale for the eligible trials.

Studies	Randomization	Allocation concealment	Blinding	Lost to follow up	ITT analysis	Baseline	Quality grading
**Nejadnik H 2010** [25]	B	A	B	A	A	A	B
**Koh YG 2012** [26]	A	A	A	A	A	A	A
**Saw KY 2013**[27]	A	A	A	A	A	A	A
**Wong KL 2013** [28]	A	A	A	A	A	A	A
**Tan YH 2013** [29]	A	A	B	A	A	A	B
**Koh YG 2014** [30]	A	A	A	A	A	A	A
**Vangsness CT Jr 2014** [31]	A	A	A	A	A	A	A
**Akgun I 2015** [32]	A	A	A	A	A	A	A
**Liang HS 2015** [33]	A	A	B	A	A	A	B
**Lv XX 2015** [34]	A	B	B	A	A	A	B
**Vega A 2015** [35]	A	A	B	A	A	A	B

Abbreviations: A, adequate, with correct procedure; B, lacks a description of the methods; C, inadequate procedures, methods, or information; ITT, intention to treat. Each criterion was graded as follows: A, studies with a low risk of bias that were scored as grade A for all items; B, studies with a moderate risk of bias with one or more grades of B; and C, studies with a high risk of bias with one or more grades of C.

### 3.2. Baseline patient characteristics

The baseline characteristics of the patients in the 11 selected publications are listed in Table 2. The trials involved a total of 582 patients with knee OA. All 11of the papers were fully published during the period from 2010 to 2015 and described nine Phase II trials. The mean ages of patients enrolled were between 32 and 57 years. Sample size ranged from a minimum of 14 to a maximum of 80 patients. The percentage of male patients ranged from 25% to 62%. In all of the trials, MSC transplantation therapy was evaluated in knee OA patients with BMSCs in 7 studies [25,28,29,31,33,34,35], ADSCs in 2 studies [26,30], peripheral blood stem cells (PBSCs) in 1 study [27], and SMSCs in 1 study [32]. The patients received cell infusions from1×10^6^ to 1.5×10^8^ cells. The injected route for MSC therapy was intra-articular injection (i.a.).

**Table 2:** Clinical information from the eligible trials in the meta-analysis.

Author and Year	Clinical trial phase	No. of patients (male) and control	Age (years, mean) and control	Follow up (months)	Control arm	Stem cell arm (Injection)	Regimens dose
**Nejadnik H 2010 (Singapore)** [25]	III	36(20); 36(18)	44.0; 42.5	25	ACI	BMSCs (i.a)	1~1.5×10^7^
**Koh YG 2012 (Korea)** [26]	II	25(8); 25(8)	54.2; 54.4	17.2	AO+PRP	AO+ADSCs (i.a)	1.89×10^6^
**Saw KY 2013 (Malaysia)]** [27	II	25(10); 24(7)	38; 42	18	AO +HA	AO+HA+PBSCs (i.a)	UK
**Wong KL 2013 (Singapore)** [28]	II	28(15); 28(14)	53; 49	24	AO +HA	AO+BMSCs (i.a)	1.46×10^7^
**Tan YH 2013 (China)** [29]	II	36(10); 36(9)	53.4; 53.8	12	AO	AO+BMSCs (i.a)	2~3×10^7^
**Koh YG 2014 (Korea)** [30]	II	21(5); 23(6)	54.2; 52.3	25.7	AO+PRP	AO+ADSCs (i.a)	4.11×10^6^
**Vangsness CT Jr 2014 (USA)** [31]	II	18; 19	46.0	24	placebo	BMSCs (i.a)	5×10^7^
		18; 19					1.5×10^8^
**Akgun I 2015 (Turkey)** [32]	II	7(4); 7(4)	32.3; 32.7	24	ACI	SMSCs (i.a)	8×10^6^
**Liang HS 2015 (China)** [33]	II	30(19); 30(18)	36.2; 35.8	16.4	AO	AO+BMSCs (i.a)	1×10^6^
**Lv XX 2015 (China)** [34]	III	40(14); 40(13)	55.9; 55.1	12	HA	BMSCs (i.a)	1.15×10^8^
**Vega A 2015 (Spain)** [35]	II	15(6); 15(5)	56.6; 57.3	12	HA	BMSCs (i.a)	4×10^7^

Abbreviations: ACI, autologous chondrocyte implantation; ADSCs, adipose-derived stem cells; AO, arthroscopic operation; BMSCs, bone marrow-derived mesenchymal stem cells; HA, hyaluronic acid; i.a., intra-articular injection; PBSCs, peripheral blood stem cells; PRP, platele-rich plasma; SMSCs, synovium-derived mesenchymal stem cells; UK, Unknown.

### 3.3. Visual analogue scale

**Fig 2:**
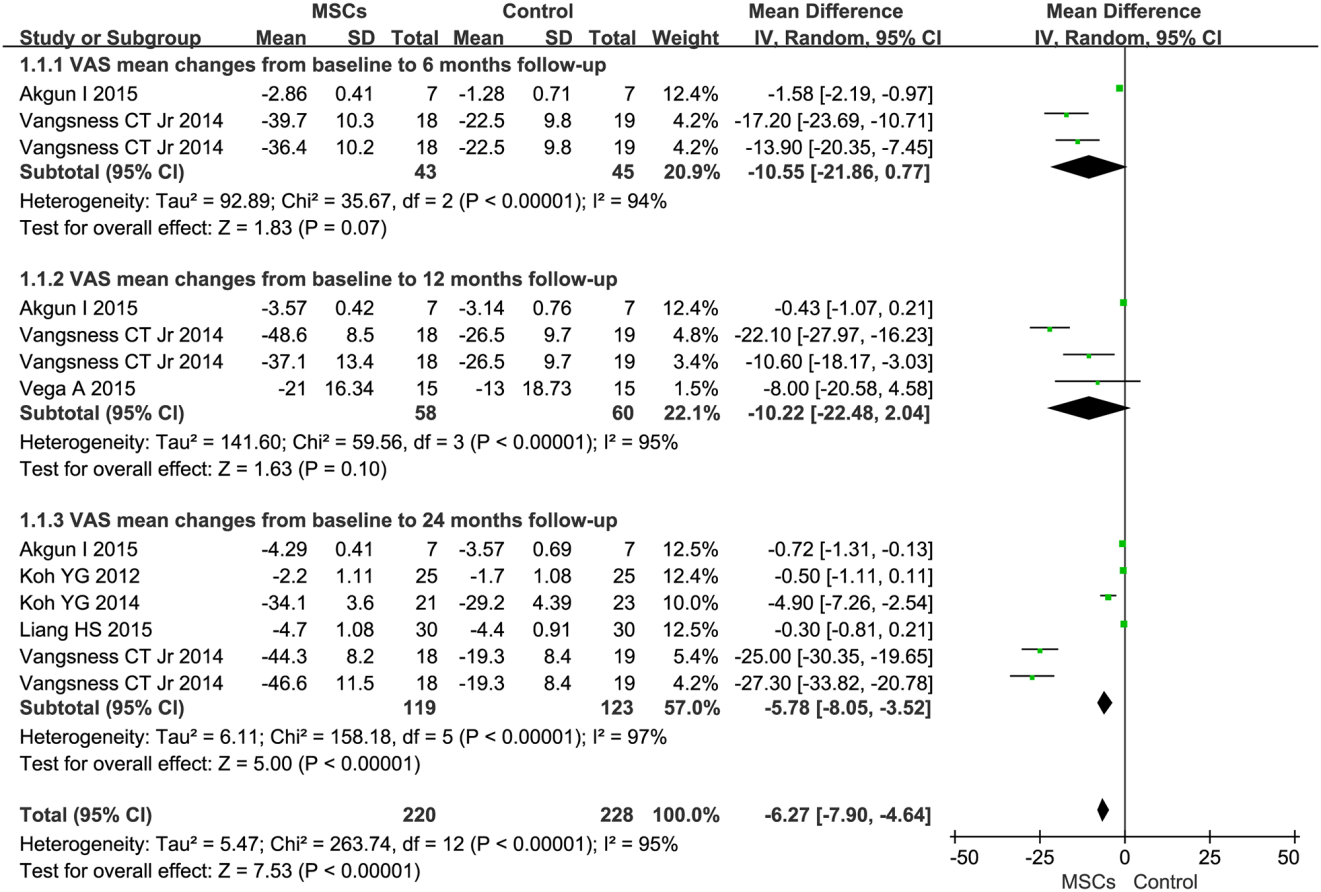
Forest plots of mean difference (MD) with 95% confidence interval (CI) in VAS between patients undergoing MSC therapy and controls at: (1) 6 months, (2) 12 months, and (3) 24 months. Random-effects models (Mantel-Haenszel method) were used. Each trial is represented by a square, and the size of the square is proportional to the information in that trial. The ends of the horizontal bars denote 95% confidence intervals (CIs). Black diamonds give the overall results of all trials.

Information on the 6-month VAS improvement was available from two trials [31,32]. These two trials contained a total of 88 patients, of whom 43 patients received MSC treatment, and 45 control patients did not receive MSC transplantation. The MD of changes in VAS of patients receiving MSC treatment was a non-significant decrease of -10.55 (95%CI -21.86–0.77, *P* = 0.07, *I*^*2*^ = 94%) compared with that of the controls. In three trials that reported 12-month VAS, the MD of changes in VAS was -10.22 (95%CI -22.48–2.04, *P* = 0.10, *I*^*2*^ = 95%). Information on the 24-month VAS improvement was available from five trials [26,30,31,32,33]. These five trials contained a total of 242 patients, of whom 119 patients received MSC treatment. The MD of changes in VAS of patients receiving MSC treatment was a significant decrease of -5.78 (95%CI -8.05- -3.52, *P*<0.00001) compared with that of the controls. Additionally, the corresponding *I*^*2*^ was 97% (Fig 2).

### 3.4. International Knee Documentation Committee

**Fig 3:**
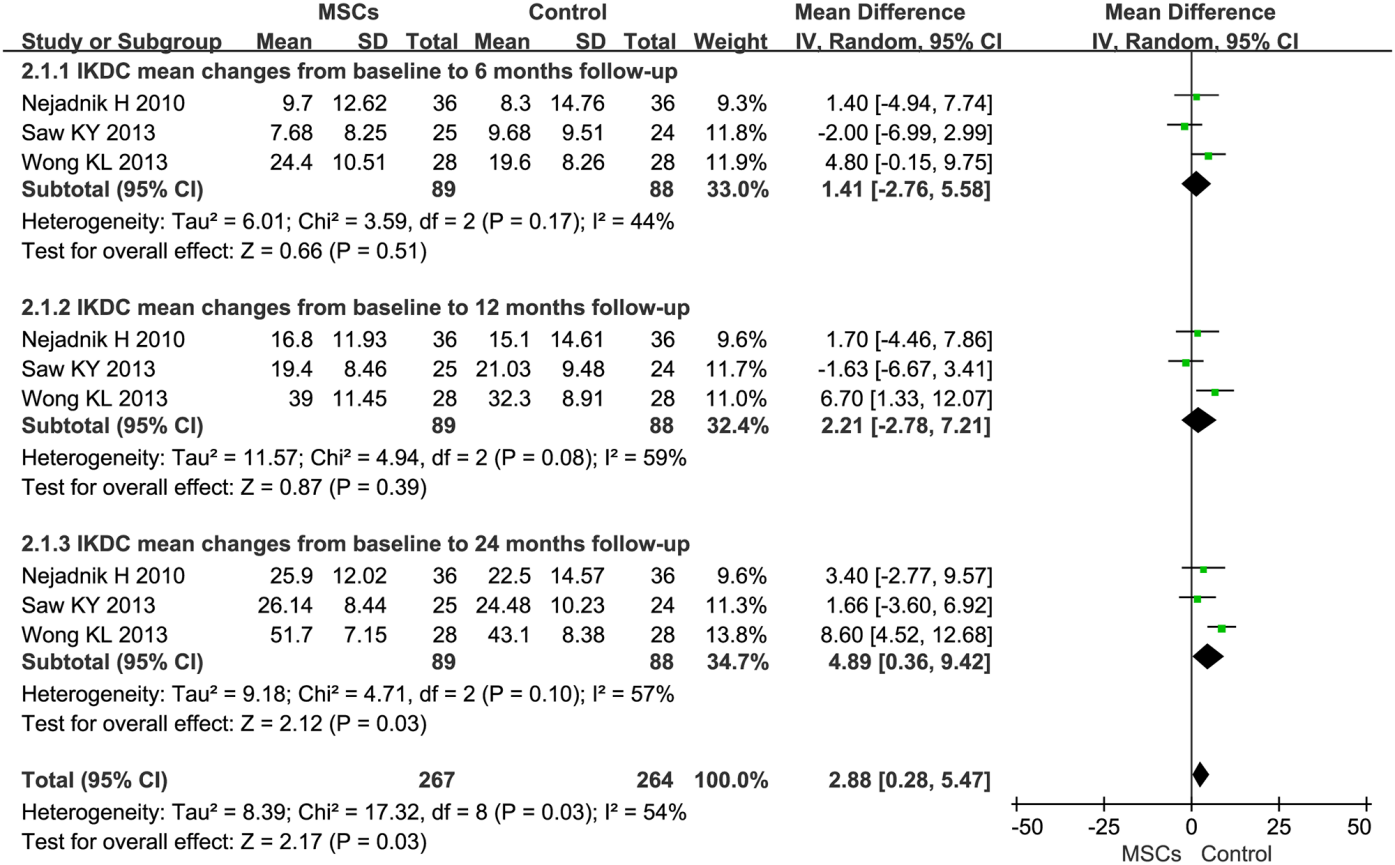
Forest plots of MD with 95% CI in IKDC between patients undergoing MSC therapy and controls at: (1) 6 months, (2) 12 months, and (3) 24 months. Random-effects models were used.

Information on the 6-, 12-, and 24-month IKDC improvement was available from three trials [25,27,28], totaling 177 patients (89 of whom received MSC treatment; Fig 3). MSC therapy led to a 6-month IKDC increase of 1.41 (95%CI -2.76–5.58, *P*>0.05, *I*^*2*^ = 44%) in patients with knee OA. The MD of changes in 12-month IKDC was 2.21 (95% CI -2.78–7.21, *P*>0.05, *I*^*2*^ = 59%). The MD of changes in 24-month IKDC was statistically significant at 4.89 (95% CI 0.36–9.42 *P* = 0.03). Additionally, the corresponding *I*^*2*^ was 57%.

### 3.5. Western Ontario and McMaster Universities Osteoarthritis

**Fig 4:**
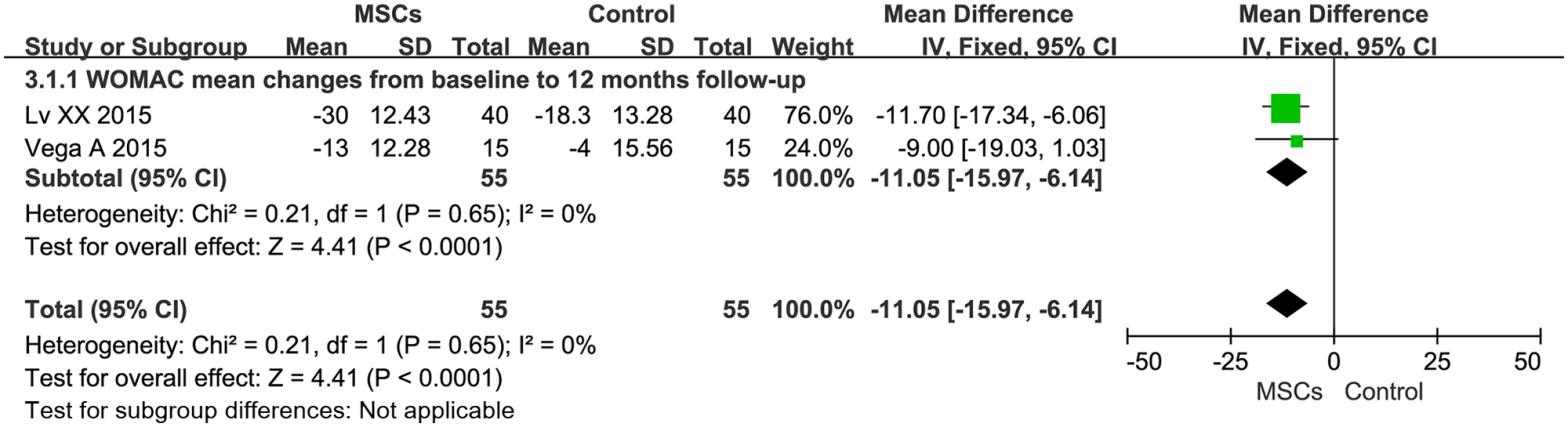
Forest plots of MD with 95% CI in WOMAC between patients undergoing MSC therapy and controls at 12 months. Fixed-effects models were used.

Information on the 12-month WOMAC improvement was available from two studies [34,35], which included a total of 110 patients (55 of whom received MSC treatment; Fig 4). The MD of WOMAC changes was statistically significant at -11.05 (95% CI -15.97- -6.14, *P*<0.0001). Additionally, the corresponding *I*^*2*^ was 0%, indicating that the degree of variability between the trials was consistent with what would be expected by chance alone.

### 3.6. Lequesne algofunctional indices

**Fig 5:**
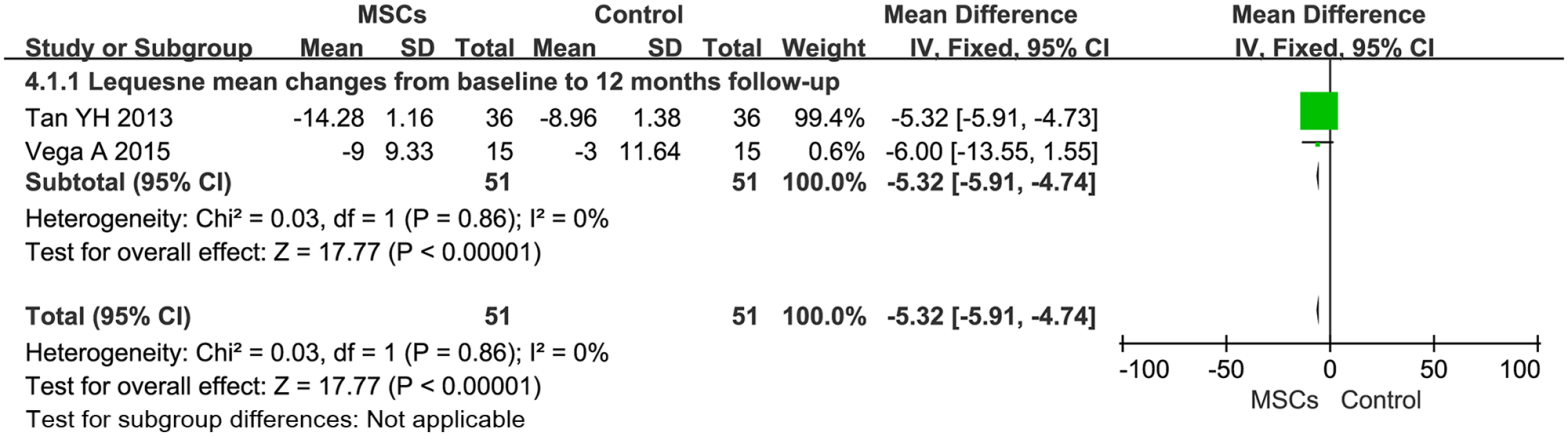
Forest plots of MD with 95% CI in Lequesne between patients undergoing MSC therapy and controls at 12 months. Fixed-effects models were used.

Information on the 12-month Lequesne improvement was available from two studies [29,35], which included a total of 102 patients (51 of whom received MSC treatment; Fig 5). The MD of Lequesne changes was statistically significant at -5.32 (95% CI -5.91- -4.74, *P*<0.00001). Additionally, the corresponding *I*^*2*^ was 0%.

### 3.7. Lysholm knee scale

**Fig 6:**
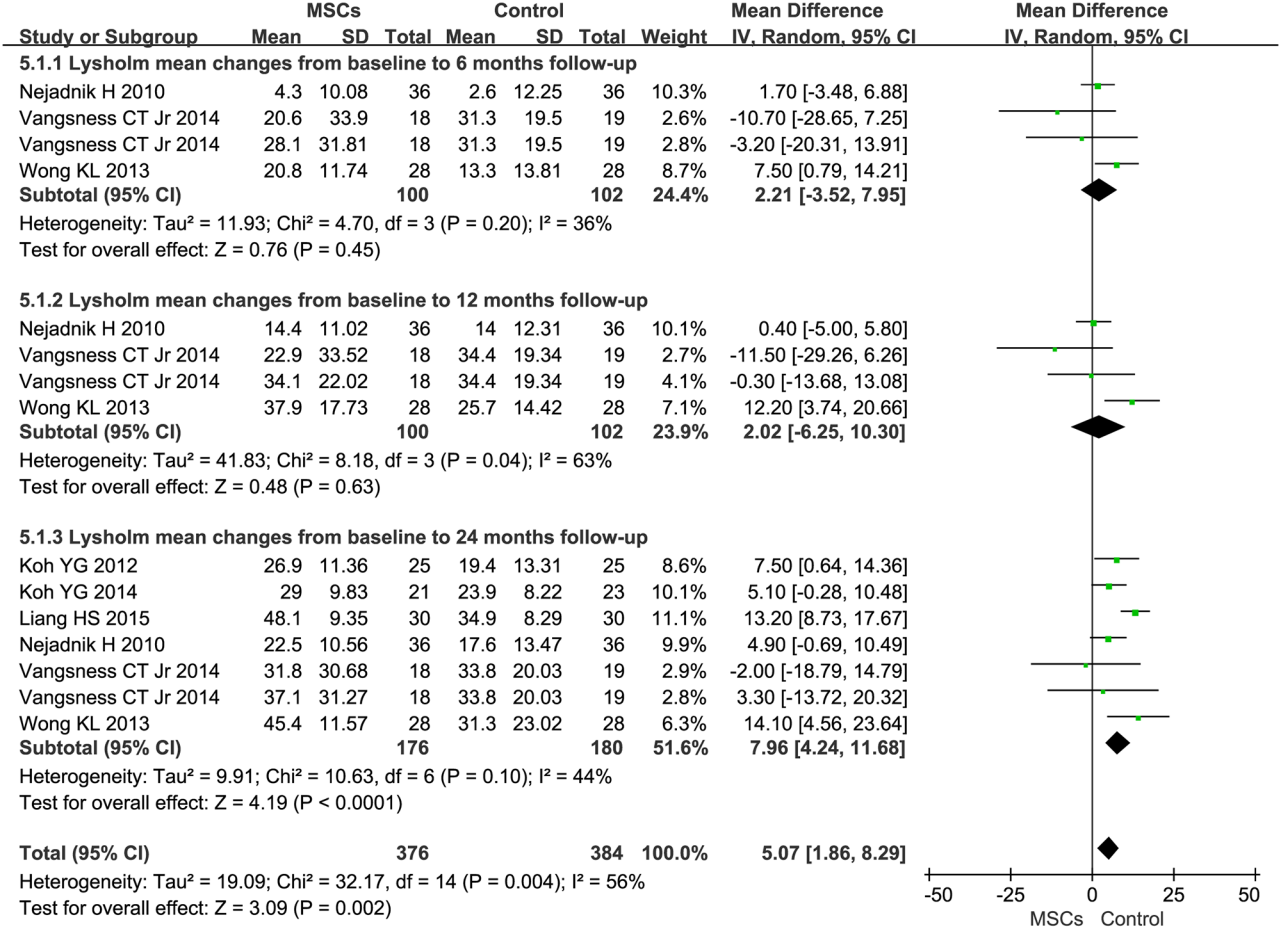
Forest plots of MD with 95% CI in Lysholm between patients undergoing MSC therapy and controls at: (1) 6 months, (2) 12 months, and (3) 24 months. Random-effects models were used.

The MD of changes in 6-month Lysholm was 2.21 (95%CI -3.52–7.95, *P*>0.05, *I*^*2*^ = 36%). In three trials that reported 12-month outcomes, the MD of changes in Lysholm was 2.02 (95%CI -6.25–10.30, *P*>0.05, *I*^*2*^ = 63%) [25,28,31]. Information on the 24-month Lysholm was available for six trials [25,26,28,30,31,33]. These 6 trials contained a total of 356 patients (176 of whom received MSC treatment and 180 controls who did not receive this treatment). The MD of changes in Lysholm was 7.96 (95%CI 4.24–11.68, *P*<0.0001, I^2^ = 44%). (Fig 6)

### 3.8. Tegner activity scale

The MD of changes in 6-month Tegner was 0.40 (95%CI -0.18–0.98, *P*>0.05, I^2^ = 68%). A pooled analysis of the data at 12 months showed that Tegner score increased significantly (MD 0.44, 95%CI 0.05–0.83, *P* = 0.03, I^2^ = 22%). A pooled analysis was performed on four trials at 24 months. The MD of Tegner changes was statistically significant at 0.46 (95% CI 0.21–0.72, *P* = 0.0004). Additionally, the corresponding *I*^*2*^ was 0%, indicating that the degree of variability between the trials was consistent with what would be expected by chance alone.

### 3.9 Toxicity and adverse reactions

The clinical trials included in this meta-analysis reported several AEs, including pain at injection site, persistent bleeding, knee swelling, warmth in the knee, fracture, difficulty moving the knee, infection in the knee, nervous system disorders, acute myocardial infarction, ileus, and small-intestine obstruction [25–35]. However, there was no statistical difference between the MSC treatment groups and controls [27,31]. Moreover, no serious AEs related to MSC implantation were developed in the 11 selected publications. Another review also reported that the application of cultured stem cells in joints appeared to be safe [36].

## 4. Discussion

Knee OA is a progressive and degenerative condition, which will remain a serious clinical problem in orthopedics unless significant advancements are made in regeneration technologies [2,37,38]. In fact, all of the currently accepted treatments are aimed at symptom control, rather than disease prevention [4,5]. MSCs are positive for the stromal cell markers CD13, CD29, CD73, CD90, and CD105 and negative for the hematopoietic markers CD31, CD34, CD45, and HLA-DR [2,39]. MSCs can inhibit the proliferation of allogeneic T cells and express low levels of major histocompatibility complex (MHCI), MHCII, and vascular cell adhesion molecule-1 (VCAM-1), so it has low immunogenicity. The self-renewing ability of MSCs and differentiation potential to become adipocytes, osteocytes, and chondrocytes has been well documented [40]. Furthermore, the homing, survival, and ability to produce extracellular matrices of MSCs in vivo have been confirmed. Previous clinical studies have shown that MSCs provide an excellent therapeutic alternative for the treatment of knee OA [41,42]. Importantly, the recent limited case series evidence has shown the cartilage volume regeneration and the disease modification after MSC injections [4,5]. MSC-based stem cell therapy could represent one of the most promising solutions for knee OA. So far, data collected from clinical trials support the following assumptions: MSCs administered into the knee adhered to and persisted on the surface of a damaged meniscus, differentiated into chondrocytes, and expressed appropriate extracellular matrix proteins (i.e. collagen I and II), resulting in a regeneration of meniscal tissue, which, with an improved meniscus, could ultimately lead to long-term chondroprotection [31]. In the present study, we performed a systemic analysis of multinational, published RCTs to assess the efficacy and safety of MSC treatment for knee OA patients using VAS, IKDC, WOMAC, Lequesne, Lysholm, and Tegner scores.

Our analysis yielded several findings. First, we demonstrated that MSC treatment could significantly decrease VAS after a 24-month follow-up (Fig 2). The estimated pooled MD showed a significant increase in IKDC after the 24-month follow-up of MSC therapy (Fig 3). WOMAC and Lequesne also showed significant decrease after the 12-month follow-up of MSC therapy (Figs 4 and 5, respectively). However, the primary endpoints did not show significant changes at other time points. The positive trend was proven to exist. Our logistic regression results showed that MSC therapy could significantly change the long-term primary endpoints of knee OA patients. The effects of MSC therapy on short-term (6-month) primary endpoints still needs to be evaluated in a larger number of patients. A recently published study by Emadedin *et al*. on autologous BMSC transplantation in knee OA patients reported that VAS and WOMAC showed a significant decrease after the 6- and 12-month follow-up [43]. Thus, a larger sample size and more elegant clinical trials are needed. Patient knee pain, stiffness, and function was assessed with the use of VAS, IKDC, WOMAC, and Lequesne. The results of our analysis indicated that MSC treatment could significantly reduce pain, improve symptoms, and improve the function of a patient’s knee OA.

**Fig 7:**
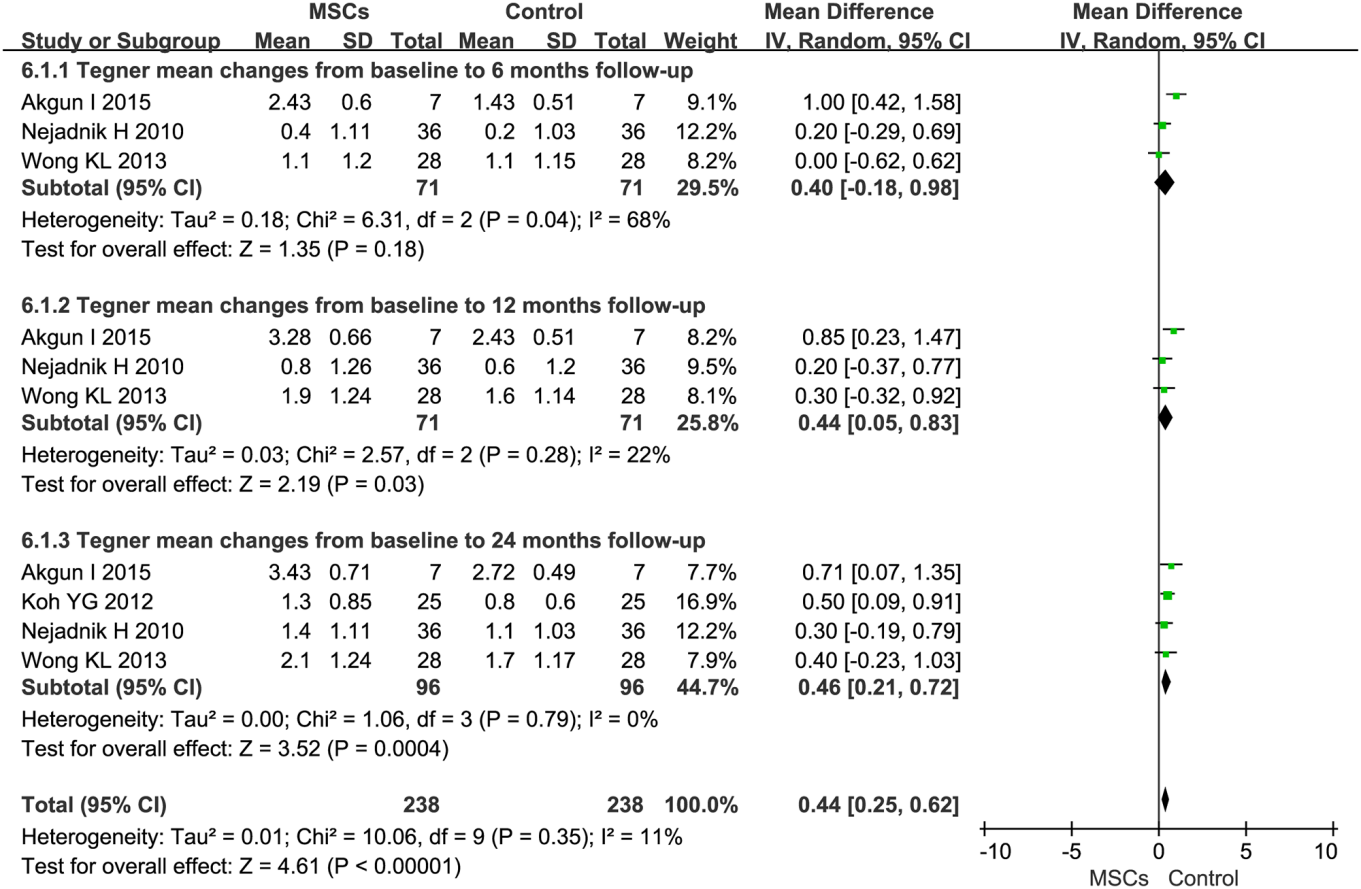
Forest plots of MD with 95% CI in Tegner between patients undergoing MSC therapy and controls at: (1) 6 months, (2) 12 months, and (3) 24 months. Random-effects models were used.

Second, the secondary outcomes of Lysholm and Tegner scores showed favorable results after MSC treatment. The estimated pooled MD showed a significant increase in Lysholm after the 24-month follow-up but not after the 6-, and 12-month follow-up (Fig 6). Our pooled analysis of the collected data showed a significant increase in Tegner after the 12- and 24-month follow-up but not after the 6-month follow-up (Fig 7). This result might be due to the small number of patients in the analysis. Thus, based on logistic regression, we concluded: MSC therapy might improve signs and symptoms of knee OA patients. Additionally, MSC therapy was shown to be safe. These scales were all subjective evaluations of knee function for patients with OA. There are, however, some reports with objective assessments of cartilage volume and quality in the eligible trials. Vangsness *et al*. reported that the cartilage volume in MSC treatment groups showed a significant decrease, observed in MRI, after the 12-month follow-up [31]. But in another trial, all MSC treatment patients showed signs of cartilage regenerationin MRI after the 12-month follow-up [27]. Vega *et al*. also reported that the cartilage quality in MSC-treated patients showed a significant improvement [35], which suggests that MSC therapy is a potential therapy for knee OA to some extent.

There are some points that may explain these results. First, transplanted MSCs could differentiate into chondrocytes directly and promote cartilage regeneration. Horie and Mizuno reported that SMSCs injected into rat knees adhered to the lesion, differentiated into chondrocytes directly, and promoted cartilage regeneration without traveling to distant organs [44,45]. Another study showed that precultured BMSCs resulted in the regeneration of meniscal tears in a rabbit model [46]. Second, transplanted MSCs have trophic and paracrine effects on the existing cartilage. MSCs could secrete an array of growth factors and cytokines, including vascular endothelial growth factor (VEGF) and hepatocyte growth factor (HGF) for neovascularization andtransforming growth factor β (TGF-β), platelet-derived growth factor (PDGF), and epithelial growth factor (EGF) to augment natural regenerative pathways [47,48]. PDGF is the most potent factor analyzed, and may be useful to promote tissue integration during cartilage repair or tissue engineering. In contrast, several studies have shown that low physiologic doses of dexamethasone could ensure that MSCs differentiate toward chondrocytes or osteogenic cells [3]. MSCs could be procured from umbilical cord, placenta, bone marrow, and fat and can easily proliferate without the use of other supportive cells. Thus, we believe that MSCs are the most suitable for knee OA treatment, considering the multiple sources and therapeutic effect.

In short, our meta-analysis demonstrated that MSC-based stem cell therapy for patients with knee OA was associated with significantly decreased VAS, WOMAC and Lequesne scores; increased IKDC, Lysholm, and Tegner scores; and low rates of AEs.

## 5. Limitations

The therapeutic effects should be interpreted with caution. The reliability of this study might be influenced by several factors. (1) *Evaluation standards* The six scales used in the selected studies are all subjective evaluations. Although patients were asked to answer all questionnaires truthfully and to the best of their ability, our study may have a moderate risk of bias. (2) *Multicenter* Eight of the selected publications in this meta-analysis were conducted in Asia, and the other three were performed in the USA, Spain, and Turkey, respectively. There is no multinational large-sample multicenter clinical research regarding MSC therapy for knee OA. Thus, the results of this analysis could not be extended to all knee OA patients across the world. (3) *Blinding and Randomization* Half of the selected studies did not use the blind method. Not all selected publications demonstrated randomization, and the sample sizes of all selected trials were not large enough. These might lead to patient, distribution, or observer biases. (4) *Heterogeneity* The high heterogeneity limits the interpretation of our results. In addition, negative trial outcomes often remain unpublished, and some good efficacy articles were excluded because they lacked appropriate control arms. Thus, the results of our meta-analysis might be misleading. We expect that our study will be useful for the design of higher quality RCTs.

## 6. Future perspectives

In the near future, MSC-based stem cell therapy could be widely used as it potentially offers substantial benefits for knee OA patients and may reduce the cost of therapy. However, there are still some unanswered questions regarding the treatment mechanism, methodology for transplanting cells, and efficacy that need to be resolved before their widespread use. First, the use of allogeneic MSCs product would have several advantages compare with autologous MSCs. Induction of humoral and/or cellular alloimmunity by allogeneic MSCs would limit their therapeutic efficacy and might provoke adverse effects [49,50]. We urgently need large RCTs utilizing standardized and established outcome scores to evaluate the clinical benefits of MSCs in cartilage repair. MRI as an objective assessment is considered to be the best way to evaluate cartilage repair. Furthermore, we still need to explore the best cell dose and culture conditions and choose the best cell infusion method for MSC therapy. In addition, the combination of MSCs with scaffolds, PRP, growth factors, and even gene therapy is also being investigated to achieve the best therapeutic effect. Moreover, the regulation of MSC treatment for knee OA is a major challenge. This requires scientists and clinicians to develop a minimum set of safety and efficacy parameters. Finally, with the continuous progress that is being made in biomedical technology, the future of MSC therapy for patients with knee OA will move toward individualized treatment.

## 7. Conclusion

Eleven selected publications regarding knee OA with 582 patients were included in the present meta-analysis. This analysis of MSC therapy in knee OA patients yielded encouraging results, with superiority in VAS, WOMAC and Lequesne scores; improvements in IKDC, Lysholm, and Tegner scores; and low rates of AEs. Hence, these results suggest that MSC therapy has great potential as an efficacious treatment for patients with knee OA. However, the safety and efficacy must be evaluated with a more rigorous, larger sample size validation before MSC therapy can be used in clinical practice.

## Supporting information

S1 Fig A funnel plot of VAS, WOMAC, Lequesne, IKDC, Lysholm, and Tegner scores (tif) generated by Review Manager Version 5.0. (TIF)

S2 Fig Language edit certification (tif). (PDF)

S1 File PRISMA 2009 checklist. (DOC)
