# Supplementary figures and images for: Clinical efficacy and safety of mesenchymal stem cell transplantation for osteoarthritis treatment: A meta-analysis
Source: PLoS One. 2017 Apr 27;12(4):e0175449. doi: 10.1371/journal.pone.0175449 (PMC5407776; doi:10.1371/journal.pone.0175449)

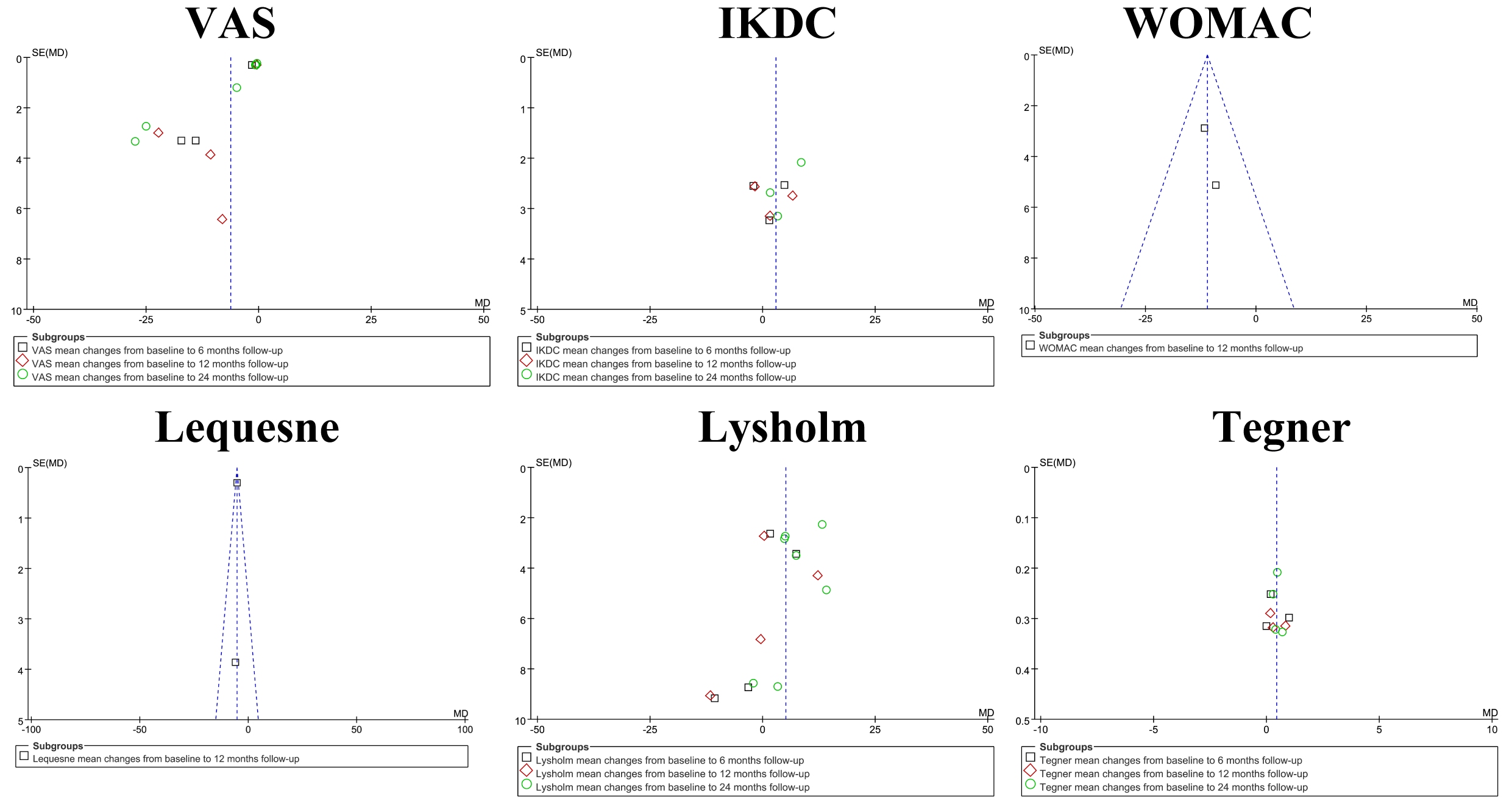

Supplement: S1 Fig — (TIF) [file pone.0175449.s001.tif]
